# General Dietary Recommendations for People with Down Syndrome

**DOI:** 10.3390/nu16162656

**Published:** 2024-08-11

**Authors:** Joanna Gruszka, Dariusz Włodarek

**Affiliations:** 1 Dieta Plus Nutritional and Dietary Counseling, 45-072 Opole, Poland; joanna_gruszka@interia.pl; 2 Department of Dietetics, Institute of Human Nutrition Science, Warsaw University of Life Science (WULS-SGGW), 159C Nowoursynowska Street, 02-776 Warszawa, Poland

**Keywords:** Down syndrome, nutrition, diet, food product groups, recommendations

## Abstract

Down syndrome (DS) is caused by trisomy of chromosome 21 and is associated with characteristic features of appearance, intellectual impairment to varying degrees, organ defects, and health problems typical of this syndrome. Studies on the frequency of consumption of food products in this group show many irregularities, in particular too low consumption of vegetables and fruits, wholegrain cereal products and dairy products, and excessive consumption of meat products and sweets. It is necessary to correct eating habits. The diets of people with trisomy 21 should be consistent with the recommendations of rational nutrition for the general population and take into account specific dietary modifications related to the occurrence of diseases and health problems characteristic of this syndrome.

## 1. Introduction

Down syndrome (DS) is caused by trisomy of chromosome 21 and is associated with features such as a characteristic appearance of the face—a flat profile, slanted eyes with skin folds, small mouth and nose—as well as short stature and intellectual impairment to varying degrees. It is also the most common cause of intellectual disability [1]. Almost all (95%) cases of trisomy 21 are the result of non-disjunction of chromosomes during meiosis. 3–4% of DS cases are translocation trisomy, where chromosome 21 or its fragment is attached to another chromosome. In 1% of cases, mosaic trisomy occurs, where some cells are normal while others contain an extra chromosome 21, and the proportion of normal to altered cells varies significantly [2].

The syndrome demonstrates many health problems that often require dietary modifications, including: congenital heart defects [3], vision and hearing defects [4,5], overweight and obesity [6,7], metabolic disorders such as hyperinsulinemia [8] and hypercholesterolemia [9,10], thyroid diseases [11,12], gastrointestinal disorders such as constipation and diarrhea, as well as congenital defects of the gastrointestinal tract [13,14,15], immune disorders [16,17], celiac disease [18,19,20], food allergies [21,22], and sleep apnea [23]. Regarding the increased cardiovascular risk in DS, it was initially believed that DS was free from atherosclerosis [24] (Rodger et al., 1977). However, newer studies show otherwise. Children with trisomy 21 had a less favorable lipid profile than their siblings, regardless of body weight [9] (Adelekan et al., 2012). Additionally, a study by Piedra et al. [25] (de la Piedra et al., 2017) found a high prevalence of dyslipidemia in a group of Chilean children with DS and suggested that children with DS should undergo regular lipid profile screenings. In individuals with DS, neurological and neurodegenerative diseases appear significantly earlier than in the general population. DS is the leading genetic cause of Alzheimer’s disease and dementia. The extra set of genes associated with this syndrome results in abnormalities in the processing of amyloid precursor protein (APP). The risk of these diseases increases with age. It is estimated that over half of individuals with DS develop Alzheimer’s disease after the age of 50 [26] (Menéndez, 2005). The prevalence of dementia ranges from 15% at age 45 to 75% at age 65 (Coppus et al., 2006; Tyrrell et al., 2001) [27,28]. Epilepsy can be a symptom of advanced Alzheimer’s disease; seizures are rare in young adults, but their frequency increases with age [29] (Lott and Dierssen, 2010; Menéndez, 2005) [26].

In therapeutic care, the importance of adapting the diet of individuals with DS not only to their needs but also to coexisting conditions and typical nutritional problems associated with this genetic defect is emphasized. There are still no nutritional recommendations for these patients, and the number of publications presenting dietary management principles for individuals with trisomy 21 is very limited. Developing dietary guidelines for individuals with trisomy 21, which would consider the complexity of problems occurring in this syndrome, seems particularly important.

## 2. Studies on the Frequency of Consumption of Food Products in People with Trisomy 21

After a thorough literature search, a total of six records were identified concerning food intake frequency studies in DS groups from PubMed, Research Gate, and Google Scholar databases (Table 1).

Several of the studies below used different questionnaires to examine the frequency of consumption of different food products. The research included different groups: children, adults, or mixed, as well as different culinary traditions, both European and Eastern cuisines. For all these reasons, the results are often not comparable.

In the study by Braunschweig and colleagues [30], the Block Food Frequency Questionnaire was used to examine the eating habits of 48 adults with DS. This questionnaire included a list of 160 products along with an example of portion sizes. The caregivers answered questions about the frequency of consumption of beverages and foods during the last week. The study by Mohamed and colleagues [31] investigated the frequency of consumption of various products by 108 children aged 5 to 12 years with trisomy 21 from Saudi Arabia. The control group consisted of the siblings of these children. In a study of Norwegian patients with intellectual disabilities [32], the frequency of consumption of products by people with Prader-Willi Williams, and Down syndromes was examined. The subjects lived both with their families and in institutionalized centers. In a study of Italian children with trisomy 21 [33], it was assessed which products they consumed most willingly. In the Polish study by Wernio et al. [34], 39 children with DS aged 9 to 18 years were examined. The study used a questionnaire on the frequency of consumption of 62 products grouped into 8 groups. In a Spanish study of 23 adults with DS [35], the questionnaire of consumption frequency and the Mediterranean Diet Prevention Questionnaire were used to qualitatively examine the diet of the respondents.

Despite researchers studying the frequency of consumption of different food groups, common conclusions can still be drawn. In the group of individuals with trisomy 21, low consumption of vegetables, fruits, and dairy products, as well as high consumption of meat and sweet snacks, was often observed.

## 3. Health Implications of an Improper Diet

### 3.1. Low Consumption of Vegetables and Fruit

Many authors indicated in the conclusions of their studies that the consumption of vegetables and fruit was low in the group of people with trisomy 21. A low intake in this group may result in less fiber and antioxidants in the diet and therefore a higher risk of developing civilization diseases. Therefore, the risk of diseases such as diabetes may be increased, the risk of which is increased not only by excess body weight and insulin resistance [8], which often occur in this population, but probably also by genetic predisposition associated with overexpression of the RCAN1 gene, which affects insulin secretion and mitochondrial function in pancreatic beta cells [36]. Another group of diseases with a higher risk are lipid metabolism disorders, which already occur in children with trisomy 21 [25], and neurodegenerative diseases, such as Alzheimer’s disease and dementia, which appear earlier in this group than in the general population [26,37,38]. A diet low in vegetables will also intensify constipation, which is often reported in this group [14], and will promote excess body weight. Vegetables are also an important source of folic acid, the deficiencies of which in children with DS are particularly at risk, due to the higher prevalence of MTHFR gene polymorphisms in mothers of children with trisomy 21 [39,40,41], according to some researchers, but also due to many reports related to the altered metabolism of one-carbon groups and thus impaired methylation in this syndrome [42].

### 3.2. Low Intake of Whole Grain Cereal Products

The low intake of whole grain cereal products is reflected in the low fiber intake. Insoluble fiber, derived from wholegrain cereals, has been linked to a reduced risk of type 2 diabetes [43,44], while soluble fiber, which is sourced, among others, from fruits, psyllium husk, and legumes, is responsible for lower postprandial glycaemia and lower insulin levels [45,46], as well as a lower glycemic index of meals [44,47]. Soluble fiber also has a significant impact on the lipid profile; studies show that it reduces the level of total cholesterol and its LDL fraction and reduces the risk of cardiac diseases [43,44,48]. All these mechanisms are important in people with trisomy 21, in whom prediabetes, type 2 diabetes, lipid metabolism disorders, as well as excess body weight are observed. Soluble fiber can also have a beneficial effect on the intestine by promoting the development of bacterial flora, which produces short-chain fatty acids (SCFAs).

The action of SCFAs is beneficial in both diarrhea and constipation, which often occur in people with trisomy 21. They affect the regeneration of colonocytes, exhibit immunomodulatory effects, lower cholesterol levels, stimulate the pancreas to secrete insulin, and activate the formation of brown adipose tissue [49,50]. Short-chain fatty acids will therefore support the maintenance of normal body weight and reduce metabolic disorders. Certainly, ensuring an adequate supply of fiber in the diet, both soluble and insoluble, is of particular importance for people with DS, especially since low fiber intake is observed in this group [33,51,52].

### 3.3. Low Intake of Dairy Products

Low consumption of dairy products was observed in studies on the frequency of consumption in people with DS, which may be related to low calcium intake in this group [33,53]. Low calcium supply is one of the elements predisposing to various complications of the skeletal system, such as disturbed bone mineralization, a predisposition to fractures, and a higher risk of the occurrence of osteoporosis. The causes of the higher incidence of osteoporosis in this population include thyroid dysfunction, sedentary lifestyle, early menopause, and epilepsy [54]. Epilepsy itself is associated with a higher risk of fractures and falls, i.e., the consequences of the disease itself, but also because of the medications used, with a four-fold increase in the risk of fractures in people who have been taking antiepileptic medicines for more than 12 years [55]. This syndrome also has a higher incidence of intracranial calcifications, leading to convulsions. The authors describing a specific case drew attention to the possible relationship between calcifications, hypocalcemia, and vitamin D deficiencies. The authors suggested that serum calcium and vitamin D levels should be checked before the implementation of anticonvulsant therapy in this type of patient [56]. In the context of trisomy 21, the problem of hypercalcemia is also described, occurring in infants and young children, usually under four years of age, and associated with impaired kidney function and calcifications occurring in them [57,58].

### 3.4. High Meat Consumption

All dietary recommendations, both American and European, recommend limiting meat consumption in the diet in favor of other sources of protein—animal and plant. On the other hand, studies on the frequency of consumption of these products show that meat is most often chosen by people with trisomy 21 from among protein-rich products, as in the general population, and other dietary sources of protein are omitted. Meat is a great source of complete protein, iron, and vitamin B12, i.e., ingredients necessary for the proper functioning of the body. Vitamin B12 deficiencies have been reported in people with DS [59]. Vitamin B12 is of particular importance in the context of hyperhomocysteinemia, which is a marker of deficiency of this vitamin, but also folic acid and vitamin B6. Hyperhomocysteinemia increases the risk of developing atherosclerosis, myocardial infarction, stroke, and also has an impact on mental health—it increases the risk of mental illness and dementia syndromes, and on the health of the mother and child by increasing the risk of miscarriages, fetal hypotrophy, preeclampsia, and neural tube defects, as well as the occurrence of Down syndrome itself [60,61,62]. Many of these health problems directly concern people with DS. High consumption of meat, and thus saturated fatty acids and omega-6 fatty acids, especially when they are not balanced with the consumption of fatty fish, i.e., polyunsaturated fatty acids from the omega-3 family, can pose a risk of developing many diseases that often coexist with trisomy 21. These include cancer, cardiac problems, and diabetes [63]. Attention is also drawn to the need to reduce meat consumption for environmental reasons, including, among others, reducing methane production and saving water [64]. Meat and other animal products are also the only dietary sources of vitamin D. The importance of vitamin D is widely described in the context of people with trisomy 21 and the health problems typical of this group. In addition to its effect on the skeletal system, the effect of this vitamin D on the immune system is also mentioned [65,66]. Its deficiency is mentioned in the context of increasing the risk of autoimmune diseases [60], cardiac diseases [67], and increasing the risk of metabolic syndrome [68]. However, it should be noted that the diet most often does not allow to cover the needs for vitamin D supply, and its adequate supplementation is essential. It is estimated that vitamin D synthesis in the skin covers 80 to 100% of the body’s vitamin D needs [69]. However, the use of SPF 15 sunscreens can reduce this synthesis by up to 99% [70].

## 4. Nutritional Recommendations for People with DS

There is a lack of comprehensive dietary recommendations for individuals with trisomy 21. Therefore, a good starting point for creating such guidelines could be the Dietary Reference Intakes (DRIs) for the general population and dietary guidelines for rational nutrition, as applied in various countries. Since national guidelines are generally designed for healthy individuals, they need to be adapted to account for the coexisting health issues commonly associated with Down syndrome.

### 4.1. Energy Value of the Diet

The development of obesity in DS is a multifactorial process consisting of difficulties with movement, resulting, for example, from hypotonia and diseases of the musculoskeletal system, reduced metabolism, predisposition to metabolic diseases, systemic inflammation, depression, but also unhealthy food choices [71,72]. It therefore seems that for people with DS with excess body weight, the diet should be characterized by a small energy deficit, always supported by a prior estimation of the actual amounts of food consumed. In addition, the patient’s communication skills, resulting from both age and degree of intellectual disability, should be taken into account, and the family or caregivers should also be involved in the recommendations [73]. On the other hand, in the case of weight deficiency, the diet should have an increased energy value and take into account the health problem that led to the underweight. These may include difficulties in swallowing meals and biting them, which will require a change in the consistency of the diet, or celiac disease, allergies, or other food intolerances, which will be associated with the use of elimination diets (Table 2).

### 4.2. Macronutrient Content in the Diet

The content of protein, fat, saturated fatty acids, carbohydrates, and fiber in the diet of people with DS should be in line with the norms for the general population [74]. However, it is possible to individually modify the macronutrients in terms of the patient’s health condition (Table 2).

#### 4.2.1. Protein

Protein sources should be varied, both plant and animal. Increased protein needs should be taken into account in malnourished people, but also often in people with excess body weight.

#### 4.2.2. Fat

The fat content in the diet of people with DS may be reduced compared to the recommendation in the case of liver and pancreatic diseases. Due to the higher risk of cardiovascular diseases and coexisting oxidative stress, the diet should take into account the limitation of saturated fatty acids and an adequate supply of monounsaturated and polyunsaturated fatty acids, in particular omega-3 polyunsaturated fatty acids.

#### 4.2.3. Carbohydrates

The main source of carbohydrates in the diet of people with DS are complex carbohydrates derived from vegetables, fruits, whole grain cereal products, as well as dry legume seeds. This will facilitate the maintenance of a proper body weight as well as reduce metabolic disorders. The type and amount of fiber may need to be modified in diseases of the gastrointestinal tract, such as inflammation of the intestines, stomach, liver, or pancreas.

For all people, it will be important to limit the intake of sugars so as not to exceed the amounts provided for in the recommendations for the general population, but also carbohydrates in total in people with excess body weight, prediabetes, and diabetes.

### 4.3. Micronutrient Content in the Diet

Increased oxidative stress is inscribed in DS because it is associated with the overexpression of several genes on chromosome 21. It is considered to be one of the most important neuropathological processes influencing cognitive deficits and neuronal changes typical of this syndrome [75]. Therefore, it seems that the diet of people with trisomy 21 should be rich in antioxidants, such as vitamins A, C, and E, and minerals, such as selenium and zinc, in order to delay the onset of civilization diseases and aging of the body. Particular emphasis should be placed on the adequate supply of B vitamins, especially folates, vitamins B6 and B12, and their supplementation due to the frequent occurrence, according to some authors, of polymorphisms of folic acid metabolism and hyperhomocysteinemia and thus impaired DNA methylation, as well as their impact on the nervous system, atherosclerosis, heart defects, and hypothyroidism [76,77,78,79]. Dietary sources of vitamin D and calcium should be taken into account. Vitamin D supplementation is also important due to its common deficiencies and calcium supplementation, especially in the population of people on a dairy-free diet (Table 2).

## 5. Dietary Guidelines in Different Countries

The cited studies on the diet of people with DS indicate the occurrence of nutritional errors, which can lead to serious health consequences. In order to improve the quality of the diet of people with DS, one can follow the generally accepted recommendations of proper nutrition, addressed to the general population. Adapting the diet of people with trisomy 21 to these recommendations will allow for a significant improvement in the quality of the diet of children and adults and will ensure an adequate supply of macro and micronutrients.

Each country creates their own nutritional recommendations, often presented as simple graphic representations, such as a plate or nutrition circle. These guidelines typically specify both the quantitative and qualitative aspects of dietary components and aim to promote health and prevent disease across all ages. Examples include recommendations from the U.S. Department of Agriculture and the Department of Health and Human Services [80], the British Eat Well Guide [81], Spanish guidelines [82], the German Nutrition Society’s nutrition circle [83], and Polish recommendations by the National Institute of Public Health [84].

Despite variations in detail, there is a global trend toward increasing fiber and bioactive compounds in the diet by emphasizing vegetables, fruits, and whole grains. Diverse protein sources, including lean meats, fish, seafood, soy products, beans, lentils, peas, nuts, and seeds, are recommended. Most guidelines suggest low-fat dairy products, except in Spain, where full-fat dairy is recommended. Vegetable fats are preferred over animal fats, and there is a universal recommendation to limit simple sugars, saturated fatty acids, and sodium. Some recommendations also address alcohol consumption; the German recommendations indicate that there is no safe level of alcohol and provide advice on physical activity, like the Spanish one.

## 6. Conclusions

Research shows that the diet of people with trisomy 21 has a number of errors regarding its composition. Too little consumption of vegetables and fruit, whole grain cereal products, dairy products, as well as excessive meat and sweets is commonly observed. In our opinion, the diet of individuals with trisomy 21 should align with the general population’s recommendations for rational nutrition and take into account specific dietary modifications related to the occurrence of diseases and health problems characteristic of this syndrome, which may adversely affect absorption, require an increase or decrease in the energy value of the diet, or require the selection of special products, e.g., gluten-free.

## Figures and Tables

**Table 1:** Studies on the frequency of consumption of various products in Down syndrome groups.

Authors	Characteristics of the Study Group	Results
Braunschweig et al., 2004 [30]	Forty-eight adults with DS	The average number of portions consumed per day in the study group was: 1.0 for vegetables, 2.8 for fruit, 1.6 for dairy products, 5.6 for bread, and 6.1 for meat. The authors found in the results that none of the participants consumed the recommended 5 servings of vegetables and fruit every day.
Mohamed et al., 2013 [31]	One hundred and eight children aged 5 to 12 years with DS and siblings as a control group	DS group: 51% of children consumed 1 to 3 portions of meat per week, the vast majority (86%) of which was poultry meat. Fish, eggs, and yellow cheese were eaten 1 to 3 times a week by the majority (80 to 86%) of the subjects. Milk was consumed 1 to 3 times a week by over half of the subjects, and pasta and rice by the majority of the subjects (89%). Only from 15% to 17% of the subjects consumed fresh vegetables and fruit in the amount of 4 to 6 portions per week.
Nordstrøm et al., 2015 [32]	Eighty-one children and adults aged 16 to 42 with Prader-Willi, Williams, and Down syndromes	DS subgroup: 33% of people with DS consumed fruit every day, fruit juices—37%, vegetables—29%. About 50% of the subjects consumed sugary drinks 3 times a week or less and 4 times a week or more often. About 70% of the subjects ate fish and pre-cooked meals 3 times a week or less. Thirty percent of the subjects ate pre-cooked meals 4 times a week or more often. The consumption of sugary drinks and ready-to-eat foods was higher among people living in communities than with families. No difference was observed in the frequency of consumption of other tested products in people with DS, living with families, and in institutionalized centers.
Roccatello et al., 2021 [33]	Thirty-four children with DS aged 6 to 16 years	Children most often chose pasta (82% of subjects), bread and its derivatives (47% of subjects), and sweets (29% of subjects). Vegetables (34% of subjects), sweets (26% of subjects), and fruit (18% of subjects) were indicated as the least liked.
Wernio et al., 2022 [34]	Thirty-nine children with DS aged 9 to 18 years	Seven percent of the subjects consumed vegetables and natural dairy products but also added sugar to their meals several times a day. Moreover, 2.5% of the subjects consumed whole grain products several times a day. Fifty-one percent of the subjects ate fruit every day, vegetable oils, nuts, and seeds—20%, sweets—7%. 43.6% consumed fatty fish at least once a week. In their conclusions, the authors found that the diet of children with DS significantly differs from the recommendations for the consumption of dairy products, vegetables, whole grains, which should be eaten several times a day, as well as fruit, seeds, nuts, which should be eaten daily, and fatty fish, which should be eaten at least once a week. The subjects preferred products that were a source of saturated fatty acids, not monounsaturated and polyunsaturated fatty acids. The subjects consumed red meat more often than fish but less often than white meat. They consumed potatoes more often than other starchy foods. Fruit was consumed more often than sweets, and fruit juices more often than sugary drinks.
Herrera-Quintana et al., 2024 [35]	Twenty-three adults with DS aged 21 to 44 years	Eighty-two percent of the subjects did not eat the recommended 5 servings of vegetables and fruit per day. In addition, excessive consumption of sweets, snacks, and red meat was observed, more than twice a week.

**Table 2:** The summary of nutritional recommendations for people with DS.

Nutrient	Recommendation
**Energy intake**	In line with the norms for the general population.
For individuals with excess body weight	Small energy deficit, always supported by a prior estimation of the actual amounts of food consumed.
For individuals with weight deficiency	Individually increased energy value with recognition of accompanying health problems causing underweight.
**Protein intake**	In line with the norms for the general population with varied sources of protein.
For undernourished individuals and those with excess body weight	Individually increased protein content.
**Fat intake**	In line with the norms for the general population with varied sources and adequate intake of monounsaturated and omega-3 polyunsaturated fatty acids.
For individuals with liver and pancreatic diseases	Individually decreased fat content.
**Carbohydrate intake**	In line with the norms for the general population with a predominance of complex carbohydrates and limitation of sugar intake.
For individuals with diseases of the gastrointestinal tract	Modification of carbohydrate sources and therefore fiber content.
**Micronutrient intake**	In line with the norms for the general population, with particular attention to dietary sources of vitamins A, C, and E, folates, vitamins B6 and B12, and minerals such as selenium and zinc.
